# Nematicidal activity of seaweed-synthesized silver nanoparticles and extracts against *Meloidogyne incognita* on tomato plants

**DOI:** 10.1038/s41598-022-06600-1

**Published:** 2022-03-09

**Authors:** Rehab Y. Ghareeb, Nihal Galal El-Din Shams El-Din, Dahlia M. El Maghraby, Dina S. S. Ibrahim, Ahmed Abdel-Megeed, Nader R. Abdelsalam

**Affiliations:** 1 Plant Protection and Biomolecular Diagnosis Department, Arid Lands Cultivation Research Institute, City of Scientific Research and Technological Applications, Borg El-Arab, Alexandria, 21934 Egypt; 2 Department of Marine Environment, National Institute of Oceanography and Fisheries, NIOF, Egypt; 3 Department of Botany and Microbiology, Faculty of Science, Alexandria University, Alexandria, Egypt; 4 Department of Nematodes diseases and Central Lab of biotechnology, Plant Pathology Research Institute, Agricultural Research Center (ARC), Giza, Egypt; 5 Department of Plant Protection, Faculty of Agriculture (Saba Basha), Alexandria University, Alexandria, 21531 Egypt; 6 Agricultural Botany Department, Faculty of Agriculture, (Saba Basha), Alexandria University, Alexandria, 21531 Egypt

**Keywords:** Analytical biochemistry, Biological models, Gene expression analysis, Genetic techniques, Immunological techniques, Nanobiotechnology, DNA, Enzyme mechanisms, Enzymes, Proteins, RNA

## Abstract

The purpose of this study was to test the nematicidal activity of extracts of two marine algae (*Colpomenia sinuosa* and *Corallina mediterranea*) and their synthesized silver nanoparticles against root-knot nematodes (*Meloidogyne incognita*) that infest tomato plants. Scanning electron microscopy (SEM) revealed that nanoparticles had aggregated into anisotropic Ag particles, and transmission electron microscopy (TEM) revealed that the particle sizes were less than 40 nm. Fourier Transform Infrared Spectroscopy (FT-IR) analysis revealed that the obtained nanoparticles had a sharp absorbance between 440 and 4000 cm^−1^, with 13 distinct peaks ranging from 474 to 3915 cm^−1^. Methylene chloride extracts and nanoparticles synthesized from both algae species were used to treat *M. incognita. C. sinuosa* nanoparticles had the highest nematicidal activity of any treatment. Furthermore, and in contrast to other treatments, *C. sinuosa* nanoparticles reduced the number of nematode galls, egg-masses per root, and eggs/egg mass, while also improving plant growth parameters. *C. sinuosa*'s methylene chloride extract was more active than *C. mediterranea*'s, and the most effective eluent of this solvent was hexane: methylene chloride: ethyl acetate (1: 0.5: 0.5, v/v/v). When applied to *M. incognita*, the third fraction of this eluent was the most effective, resulting in 87.5% mortality after 12 h and 100% mortality after 24 and 72 h of exposure. The presence of seven bioactive constituents was discovered during the analysis of this fraction. In conclusion, the silver nanoparticles synthesized from *C. sinuosa* could be used as alternative chemical nematicides.

## Introduction

Plant-parasitic nematodes (PPN) cause significant damage to the majority of agricultural crops in tropical and subtropical regions^1–3^, with annual losses estimated to be $100 billion worldwide^4,5^. The root-knot nematode (*Meloidogyne* spp.) is common and affects a wide range of crops^6,7^. More than 3000 host species cause serious damage to most agricultural crops around the world^5,7^. *Meloidogyne incognita*, *Meloidogyne javanica*, *Meloidogyne arenaria*, and *Meloidogyne hapla* are the most damaging nematodes for crops, infecting over 3000 host species^5,9^. Plant-pathogenic nematodes (including root-knot nematodes) reduce crop yield by 8.8% in developed countries and up to 14.6% in tropical and subtropical regions. They infest a wide range of important crops and are more damaging to vegetables than to others^8,9^. *M. incognita* can cause crop failure in the absence of effective control. The tomato (*Solanum lycopersicum*) is the world's most important vegetable crop, but it is frequently attacked by *Meloidogyne* spp*.*, limiting fruit yield quantity and quality^10,11^. Tomato yield losses due to root-knot nematodes have been estimated to be up to 61.0%^12^. Several studies have found that the damage caused by *Meloidogyne* spp*.* on various tomato cultivars in pot, microplot, and field experiment conditions varies depending on which species is causing the infection^13^, For tomatoes infected with *M. incognita*, yield losses of 22%–30% and damage ranging from 32 to 40% have been reported^14^. Chemical pesticides are commonly used to control PPNs. Notably, excessive use of such pesticides, known as "nematicides," has had a negative impact on the environment and human health^15^. Furthermore, the widespread use of nematicides has resulted in an increase in nematode pesticide resistance.

Nanotechnology has recently been used to successfully manage pest-infected plant crops^2,5,16–21^. Furthermore, the use of silver nanoparticles (Ag NPs) has clarified anti-nematode effects^22^. However, chemical synthesis of silver nanoparticles is frequently prohibitively expensive, necessitates the use of toxic and hazardous chemicals, and poses potential environmental risks^23^. They may also pose environmental and biological risks. As a result, a large number of recent studies have concentrated on the viability of biological synthesis of environmentally friendly, non-toxic nanocomposites^24,25^.

The use of biological materials for the synthesis of nanoparticles includes plant extracts, fungi, bacteria, and seaweed, which avoids the use of toxic chemicals and thus presents a number of advantages over chemical synthesis, including eco-friendliness and compatibility for pharmaceutical manufacturing and other biomedical applications^5,24,26,27^. Among all species of algae, Chlorophyta, Phaeophyta, and Rhodophyta are considered the most important and major groups^28^. These groups contain a wide range of seaweeds (marine algae) with unexplored biochemical compounds such as carotenoids, dietary fibers, agar, acids, carotenes, alkaloids, fatty acids, and phenolic compounds, all of which could be sources of novel pest control agents^29^. Seaweeds also contain antibiotics like bromo-phenols, tannins, phloroglucinol, and terpenoids, which may have anti-nematode activity^32^. Antibiotics, such as bromo-phenols, tannins, phloroglucinol, and terpenoids have antinematode activity^30^. Alginate, a type of crystallizing agent derived from marine brown algae, is widely used in the food industry, medicine, and plant-pest biocontrol^31^. The main benefits of alginate preparations are their non-toxicity, rapid degradation rates, and the release of microorganisms into the soil^32^.

Notably, alginates extracted from *Colpomenia sinuosa* significantly reduced reproduction of *M. javanica* infecting eggplants (*Solanum melongena* L.) and increased plant host growth compared to untreated, infected plants under greenhouse conditions^33^. *Corallina sp.*, a red seaweed, could be investigated as a potential source of bioactive molecules such as minerals, saturated fatty acids, sulfated galactans, and carrageenan, all of which have nematicidal and antimicrobial activity^34^. The nematicidal activity of *Corallina officinalis*, *Corallina mediterranea*, and *Ulva fasciata* against the root knot nematode *M. incognita* was demonstrated. Nanotechnologies have recently advanced in biology, medicine, pharmacology, and agriculture. It boosted the use of green silver nanoparticles as a novel method of controlling root knot nematodes^35,36^. According to Khan et al.^37^ various seaweeds have significant nematicidal activities such as inhibiting egg hatching, increasing larval mortality, and reducing root-knot disease. Furthermore, nanoparticles (NPs) have distinct physicochemical properties and the potential to improve plant metabolism^38^. As a result, the current study was carried out to assess the efficacy of *Colpomenia sinuosa* and *Corallina mediterranea* extracts, as well as synthesized green Ag NPs, for their nematicidal activity against second-stage juveniles (J2S) of *M. incognita* infecting tomato crops. Furthermore, the study aimed to identify the bioactive compounds with the highest nematicidal activity from the two algal extracts studied.

## Materials and methods

The chosen tomato plants are officially collected from the in-house Department, Arid Lands Cultivation Research Institute, City of Scientific Research and Technological Applications, Borg El-Arab, Alexandria, Egypt. This Farm is solely for research and development only.

### Study area

Algal samples were collected in June 2015 from two stations on the coast of Alexandria, Egypt, Abu-Qir (31° 19′ 26″ N, 30° 3′ 41″E) and Gleem seashore (31° 15′ 28″ N, 29° 57′ 28″ E).

### Collection of macroalgae

Algal samples were collected by hand at the sub-littoral zone (0.5–1 m depth), washed in seawater at the sampling site to remove adhered sediments and impurities, and then separated into polyethylene bags. The samples were kept cold in an ice box at 4 °C. On the same day, a quick rinsing of the collected algae with tap water was performed in the laboratory to remove any remaining impurities and epiphytes. A microscopic examination of a whole collected mount of each algal species was carried out, and morphological identification was performed as previously^39,40^. The first alga found in Abu-Qir was identified as *Colpomenia sinuosa* (Mertens ex Roth) Derbes et Solier, which belongs to the Phaeophyceae class, order Ectocarpales, and family Scytosiphonaceae. *Corallina mediterranea* Areschuog, a member of the class Rhodophyceae, order Corallinales, and family Corallinaceae, was identified as the second alga collected from Gleem's seashore. Approximately 250 g of each species were air dried to constant weight at room temperature (25 °C). Consequently, the dried alga was ground into a fine powder in an electric blender.

### Source of root knot nematodes

Nematode inoculum was extracted from pure cultures of *M. incognita* grown on black nightshade, in the greenhouse of Alexandria's City of Scientific Research and Technological Application, *Solanum nigrum* Linn. (Solanaceae). The extracted galled roots were collected and washed with tap water to remove the adhering soil particles, and the egg masses in the galls were collected with a needle under stereoscopic microscope (LABOMED; Labo America, Inc. USA). Egg masses were incubated in petri dishes with distilled water for 48 h at room temperature (27 ± 2 °C) to induce hatching. Active J2S was collected after hatching. Approximately 3000 root knot nematode J2s were inoculated per pot into one-month-old tomato plants cv. Alisa, which were planted in 2.5 kg of sterile sandy/clay soil mixture in the greenhouse at 27 ± 2 °C^41,42^.

Thirty-five days after nematode inoculation, nematode eggs were extracted from galled roots by washing and cutting the roots into 1 cm strips, followed by shaking the root strips for 3 min in 1 L of 0.5% of sodium hypochlorite solution (NaOCl)^43,44^. The resulting egg suspension was sieved through 200 and 500 mesh sieves. In 100 ml plastic beakers, nematode eggs retained by the 500 mesh sieve were collected. Nematode eggs were then left to hatch in sterile distilled water at 26 ± 3 °C, and newly hatched J2S were collected. Freshly hatched J2S collected were used as nematode inoculum.

### Morphological identification of root knot nematode

After Meena et al.^45^, ten mature Meloidogyne spp. females were removed from the root tissue using forceps. Females were separated from egg masses and placed in a drop of warm lactophenol on a clear glass slide to be examined under a light microscope for perennial pattern identification^46^.

### Preparation of the macroalgae extracts

The efficacy of different analytical-grade organic solvents for performing algal extracts from the two collected species*, C. sinuosa* and *C. mediterranea*, was compared. Fractionation was used for extracts^47^. Five gram powder from each dried algal species were extracted with 50 ml n-hexane (1:10 w: v) and shaken at 150 rpm overnight (Hermle Labortechnik GmbH, Germany). The extracted solution was centrifuged at 10.000 g (Hermal Labortechnlk, Gmbh. Germany) for 15 min to collect the supernatant. The extract was separated from the alga, using filter paper (GVS, 125 mm). Following hexane extraction, the seaweed samples were air dried in a ventilated area at ambient temperatures (25 ± 2 °C) until a constant weight was achieved. The air-dried residue was then extracted three times, once with each organic solvent (methylene chloride, ethyl acetate and finally n-butanol), following the same procedure as used in the first extraction. The four supernatants obtained from the extraction of each algal species were used for separate bioassay tests for *M. incognita;* the methylene chloride extract exhibited the highest activity for both algae. As a result, five grams of each species' algal powder dry weight were dispersed in 100 ml methylene chloride and shaken at 150 rpm at 45 °C overnight. Filtered through a Millipore filter (0.2 μm) and stored at -20 °C for further study.

### Biosynthesis of Ag NPs using macroalgae extract

Silver nanoparticles (Ag NPs) were biosynthesized as described by Azizi et al.^48^ with minor modifications. For both *C. sinuosa* and *C. mediterranea*, 100 ml of algal extract was mixed for one hour at 40 °C with 100 ml of aqueous solution (1 mM) AgNO3, then allowed to stand for one hour at room temperature (25 °C). The reaction's color changed from transparent yellow to dark brown, indicating the formation of Ag-NPs. The synthetic reaction was completed in 2 h. The initial pH of the solution was approximately 7.5, but by the end of the reaction, it had dropped to 5.6. The dark brown solid product was collected by centrifugation at 11.000 g for 12 min and washed five to ten times with distilled water. The final pellet was dried at 35 °C. The dried sample was mixed with a few drops of ethanol, ground into powder and stored for further analysis.

### Purification of synthesized silver nanoparticles

Biosynthesized silver nanoparticles were purified with distilled water and 70% ethanol by repeated centrifugation at 5.000 g for 20 min.

### UV–Vis spectrophotometer

The UV–visible absorption of the algal extract and AgNO3 mixtures was measured at room temperature using a T60 Visible Spectrophotometer (PG Instruments Limited). The absorbance of silver nanoparticles was monitored at O.D. of 450 nm.

#### Characterization of Ag-NPs using scanning electron microscope (SEM)

The changes in color and morphology of the Ag NPs were visualized using a scanning electron microscope (SEM). The synthesized AgNPs, which were harvested by centrifugation at 8.000 g for fifteen minutes at 4 °C, washed with absolute ethanol and fixed with 2% glutaraldehyde followed by 1% osmium tetroxide (OsO4). After fixation, the samples were washed with absolute ethanol and dehydrated in increasing ethanol concentrations (50, 75 and 100%). The dried fixed Ag NPs were then coated with a thin layer of gold. The average particle size of Ag NPs was determined by measuring the size of randomly selected particles in each sample using the SMILE VIEW software and a JEOL JSM-6490 (JEOL, USA).

#### Characterization of Ag NPs using transmission electron microscopy (TEM) and EDX analysis

TEM samples of synthesized Ag NPs were prepared by dispersing small quantities of the dried sample in distilled water and depositing a few drops of the resulting suspension on a copper grid (Field Emission Transmission Electron Microscope, JEOL-JEM-2100F).

#### Characterization of Ag NPs using Fourier transform infrared spectroscopy (FT-IR)

The functional biomolecules present in the algae that could be responsible for the Ag NPs formation was examined and characterized using FT-IR spectrometer (FTIR-8400S, Shimadzu, Japan). To determine the composition of the dried Ag NPs, they were compressed into thin pellets with potassium bromide (KBr) powder and scanned at wavelengths ranging from 400 to 4000 nm.

#### Characterization of Ag NPs using XRD

X-ray powder diffraction (XRD-7000 model, Shimadzu, Japan) using CuKα radiation (λ = 1.54060 Å) was performed to determine the crystalline structure of the Ag NPs using a stepwise scanning method (2θ range from 5–80°) with a scan speed of 4θ/min. The average crystal size (D) was recorded following Sallam et al.^3^.

### Nematicidal activity on second-stage juvenile mortality

A laboratory experiment was carried out to assess the nematicidal effect of algal extracts and algal synthesized Ag NPs on *M. incognita J2* mortality. Second-stage juveniles were treated with algal extract or different concentrations of algal-derived AgNPs (S = 9 ml AgNPs + 1 ml nematode suspension, S/2 = 4.5 ml AgNPs + 4.5 ml distilled H2O + 1 ml nematode suspension and S/4 = 2.25 ml AgNPs + 6.75 ml distilled H2O + 1 ml nematode suspension). The bioassay was conducted in 10-well cell culture plates, with approximately 30 freshly hatched J2s per ml representing each treatment. In two sets of assays, each replicated five times, distilled water (9 ml distilled H2O with 1 ml nematode suspension) was used as control and Nemacur 400 EC (9 ml distilled H2O + 1 ml nematode suspension + 10 µl Nemacur 400 EC ) was used as a reference nematicide. The plates were incubated at 25 ± 2 °C for 12, 24 and 72 h after treatment, and the mortality of J2s was recorded. The nematodes were considered dead if they appeared motionless in plain water^49,50^. The percentage of mortality was calculated according to Karthik et al.^51^.

$$Mortality \% = \left[ {\frac{{\left( {Total number of alive J2S in control - No. of alive J2S in treatment} \right)}}{No. of total alive J2S in control}} \right] \times 100$$

### Nematicidal activity of two macroalgal algal extracts and their synthesized Ag NPs against M. incognita in vivo

To investigate the nematicidal activity and the impact of algal extracts on tomato growth, tomato seedlings from 45-day old cultivar Alisa were sown in 20 cm diameter sterilized pots filled with autoclaved (121 °C for 1 h) and ventilated sandy: clay soil (1:1 v/v) in a greenhouse at the Faculty of Agriculture, Alexandria. The seeds were obtained from Department of Vegetable Sciences, Faculty of Agriculture, Alexandria University, Egypt.

Six treatments with 10 replicates each were applied in this experiment. In all treatments, the pots were inoculated with 2,000 M*. incognita* J2s and eggs^52^. The treatments were as follows: First, a negative control with only *M. incognita* inoculum; second, a positive control with Nemacur 400 EC (1 ml/pot) applied to plants previously inoculated with *M. incognita*; third and fourth, simultaneous application of *M. incognita* inoculum and *C. sinuosa* extract (40 ml/pot) and algal-derived AgNPs (100% conc.); and fifth and sixth, simultaneous application of *M. incognita* inoculum and *C. mediterranea* extract (40 ml/ pot) and synthesized AgNPs (100% conc.). Pots were watered three times a week with approximately 300 ml of fresh water.

The pots were arranged in a greenhouse in a randomized-block design. 60 days after nematode inoculation, the plants were harvested and thoroughly washed to remove the surrounding soil. Fresh and dry weights of root and shoot systems, the numbers of nematode root galls, egg masses, and eggs/egg mass were all measured. Egg masses were stained for about 15 min with phloxine B stain (0.15 g/l tap water) then washed with tap water^48^.

### Thin layer chromatography (TLC)

Because *C. sinuosa*'s methylene chloride extract performed better in the bioassay than *C. mediterranea*'s, it was applied to a plate of silica gel (60–120 mesh) thin-layer chromatography. The thin- layer chromatography of a purchased precoated silica plate was established by selecting a small area of 1.5 cm on the plate and adding a few drops of different methylene chloride eluents leaving at least 1 cm between each small area. The flow rate of the active material was determined using different eluent systems. The active material was eluted using the following eluents, each with a different degree of solvent polarity, Hexane: methylene chloride (9:1 v/v); hexane: methylene chloride: ethyl acetate (1: 0.5: 0.5 v/v) and hexane: methylene chloride: ethyl acetate (2.5: 1: 0.5 v/v). In each case of the chromatograms, the solvent front was marked, and spots were identified with pencil, which were observed under a UV lamp (CAMAG Model, short wavelength 254 λ, high wavelength 365λ), and the retention factor (Rf.) was calculated. The migrating spots of the detected active material were visualized by using UV lamp (UVS-II).

### Preparative thin layer chromatography

Preparative thin-layer chromatography was applied to a plate of silica gel (60–120 mesh) to fractionate the most effective eluent of methylene chloride; Hexane: methylene chloride, namely ethyl acetate (1: 0.5: 0.5 v/v).

### GC–MS analysis of methylene chloride crude extract

The most effective methylene chloride eluent fraction (Hexane: methylene chloride: ethyl acetate; 1.5: 0.5: 0.5 v/v) from *C. sinuosa* was further analyzed using gas chromatography-mass spectrometry (GC–MS) and its chemical constituents were identified^53,54^. The analyses were performed using an Agilent 7693 series GC equipped with an OV-5 capillary column (length 30 m $\times$ diameter 0.25 mm $\times$ film thickness 0.25 µm, Ohio Valley Specialty Chemical, Inc.) and an Agilent 5975C network selective mass detector. The extract was prepared by soaking the dry algal material in the eluent over three consecutive soakings (1:10 w/v) and the filtrate was subjected to GC–MS analysis (Perkin Elmer), with the primary temperature set to 90 °C for 1 min, and 300 °C for 30 min. The sample (injection volume of 1 μl) was injected into the splitless mode for 61.87 min total run time. The mass spectrometer was set to electron impact (El) mode at 70 eV, with a scanning range of 60–600 m/z. By comparing the GC–MS peaks with standard retention times, the chemical constituents of the methylene chloride eluent fraction were discovered and the mass spectra obtained were associated with those available in the Mass Spectral Library NIST 2015^55^. The percentage of each component was estimated as the ratio of the peak area to the total chromatographic area^56^.

### Statistical analysis

Data was statistically analyzed using analysis of variance (ANOVA), and differences between means were tested for significance at p.05 using the revised LSD test and the statistical analysis system SAS^57^.

## Results

### Morphological observations

**Figure 1:**
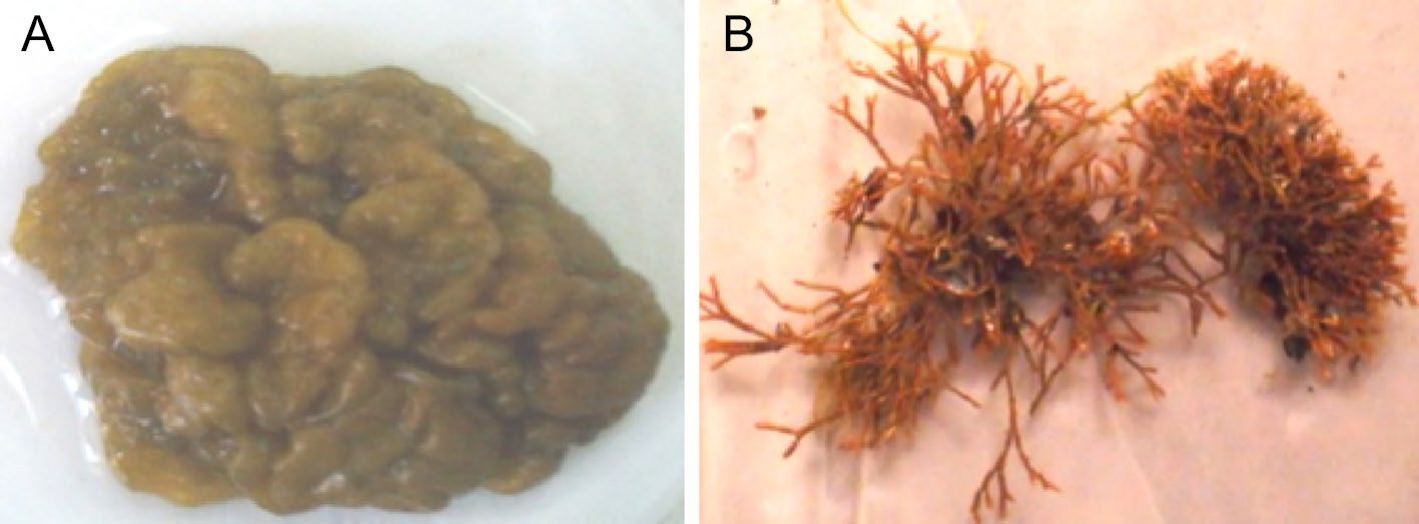
(**A**) *Colpomenia sinuosa* collected from Abu-Qir and (**B**) *Corallina mediterranea* collected from Gleem in (2016) (photographs were tacken by the Prof. Dr. Nihal Galal El-Din Shams El-Din).

The algal samples were made up of two different species (Fig. 1A,B). The first, *Colpomenia sinuosa* (Mertens ex Roth) Derbes et Solier, is a member of class Phaeophyceae, order Scytosiphonales, and has one family (Scytosiphonaceae). The second, *Corallina mediterranea* is a member of class Rhodophyceae, order Corallinales, family Corallinaceae.

### Characterization of Ag NPs

UV–Vis spectroscopy, FT-IR, SEM, and TEM were used to characterise the biosynthesized eco-friendly *C. sinuosa* (NPs).

#### UV–Vis spectral

**Figure 2:**
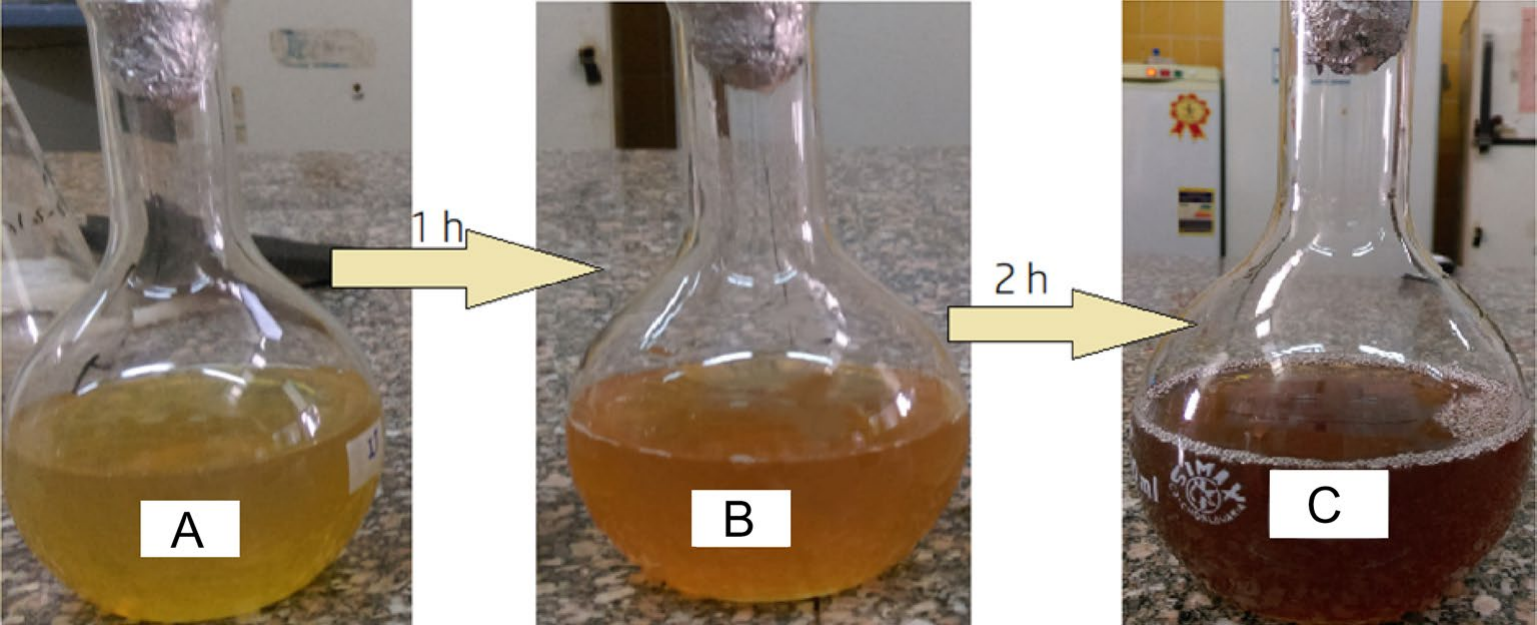
Color change during the bio-reduction of AgNO3 into AgNPs using *C. sinuosa* extract: (**A**) *C. sinuosa* extract before synthesis, (**B**) AgNO3 solution after adding *C. sinuosa* extract after one hour, (**C**) synthesized silver nanoparticles in dark brown colour solutions after two hour.

It is well known that the presence of AgNPs is indicated by a brown coloration. Because of surface plasmon resonances (SPR) within the particles, aqueous solutions containing AgNPs appear clear, yellowish, brown, and dark brown. AgNPs were produced in our study by exposing *C. sinuosa* extract to an AgNO3 solution. During 2 h of incubation, the Ag ions were completely reduced (Fig. 2).

**Figure 3:**
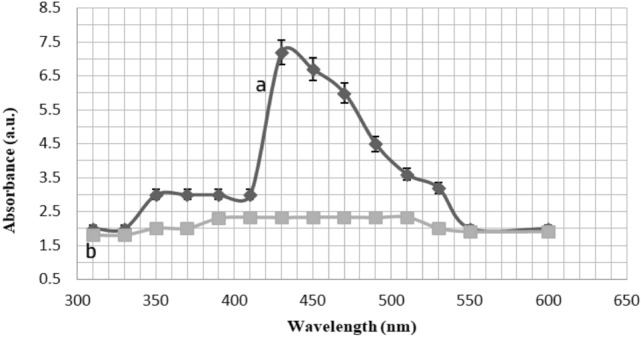
UV–visible rang spectra of (**a**) Ag NPs synthesized from *C. sinusa* extract and (**b**) *C. sinusa* extract.

Visual observation confirmed the formation of silver nanoparticles. At 480 nm, a distinct peak in the UV–vis absorption spectrum of AgNPs biosynthesized by *C. sinuosa* extract was detected, indicating the presence of SPR (Fig. 3). After 2 h of reaction, a distinct peak at 430 nm was detected in the UV–vis absorption spectrum, which steadily increased in intensity as reaction time increased. In addition, the UV–visible absorption spectra shown in Fig. 3. This absorbance peak indicated the presence of Surface Plasmon Resonance (SPR). The formation AgNPs was very fast (2 min for algal extracts and 2 h for powder) and they remained stable in colour for a long time at room temperature.

#### FT-IR spectra

**Figure 4:**
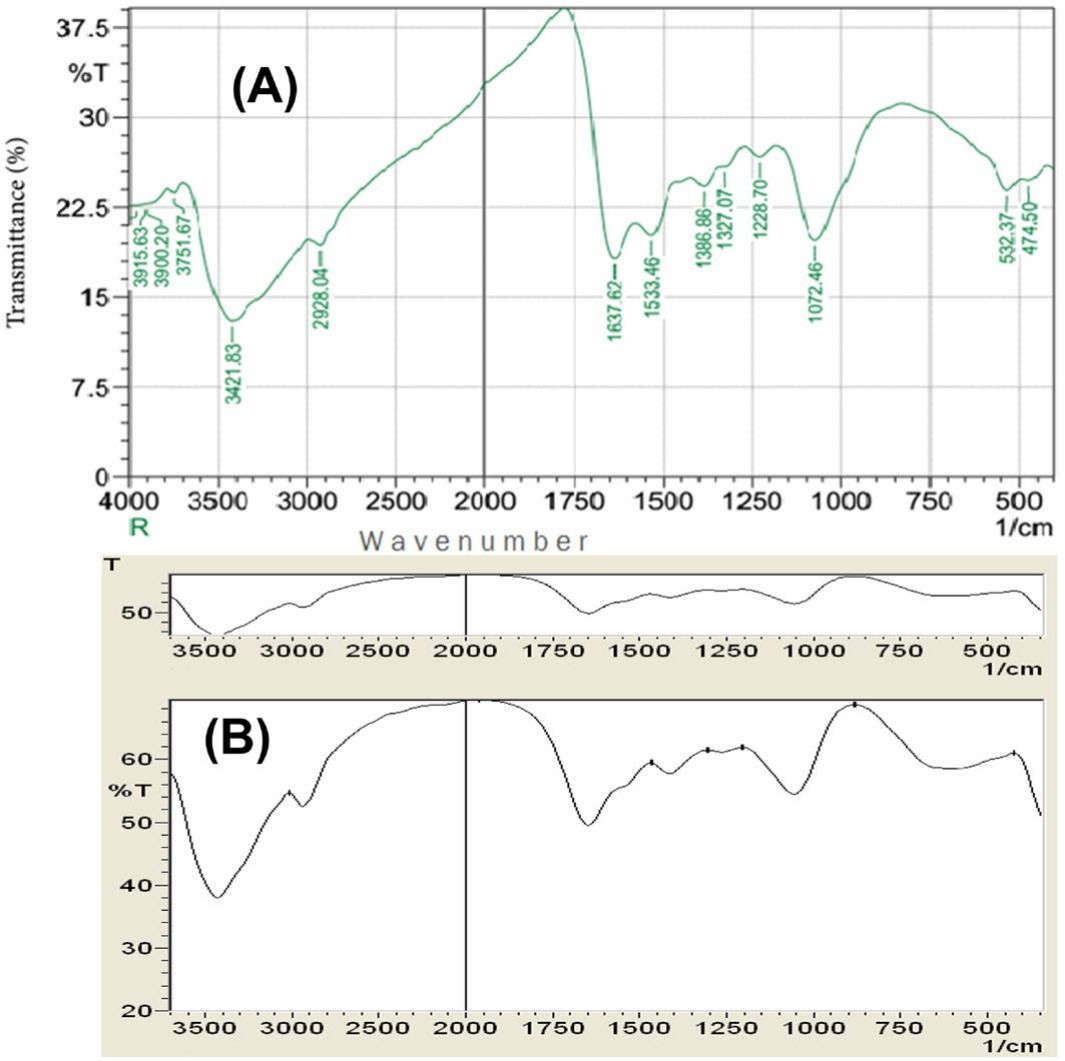
Fourier-Transform Infra-Red spectra (FT-IR) spectra shows (**A**) the functional groups associated with Ag NPs synthesized by using *C. sinuosa* extract and (**B**) *C. sinuosa* extract.

The Fourier-Transform Infrared spectra (FT-IR) were used to characterize the biomolecules for silver ion reduction in *C. sinuosa*-derived AgNPs. Figure 4a,b revealed the presence of several distinct peaks: 3915, 3900, 3751, 3421, 2928, 1637, 1533, 1386, 1327, 1228, 1072, 532, and 474 cm − 1. Peaks at 3915 cm^−1^ and 3900 cm^−1^ correspond to an O–H stretching vibration, indicating the presence of an alcohol group, while the band at 3751 cm^−1^ corresponds to a C-H stretching band. The broad spectrum at 3421 cm^−1^ indicates the presence of alcohol and phenol due to the strong stretching vibrations of an O–H functional group. The presence of C–H stretching vibrations in the broad spectrum of the peak at 2928 cm^−1^ suggests the presence of alkanes or possibly a secondary amine. The presence of N–H bent primary amines is revealed by the band at 1637 cm^−1^ in the spectra, which corresponds to (–NH–C=O). The peak at 1533 is caused by N–O stretching of aromatic nitro compounds, the peak at 1386 cm^−1^ is caused by symmetric carboxylate stretching, and the peak at 1327 is caused by N–O asymmetric stretching and reveals the presence of a nitro compound's functional group. The peaks at 1228 cm^−1^ and 1072 cm^−1^ were attributed to aromatic ether C–O or C–O–C stretching. On the other hand, the very weak bands at 474 and 532 cm^−1^ indicated presence of alkyl halide vibrations. Accordingly, the FT-IR results showed that the biosynthesized Ag-NPs were successfully synthesized and capped with bio compounds found in the *C. sinuosa* extract using the bio-reduction method. Following that, the *C. sinuosa* aqueous extract spectrum was consistent with a sulphated polysaccharide, which is relevant for its antioxidant and antimicrobial activity (Fig. 4a,b).

**Figure 5:**
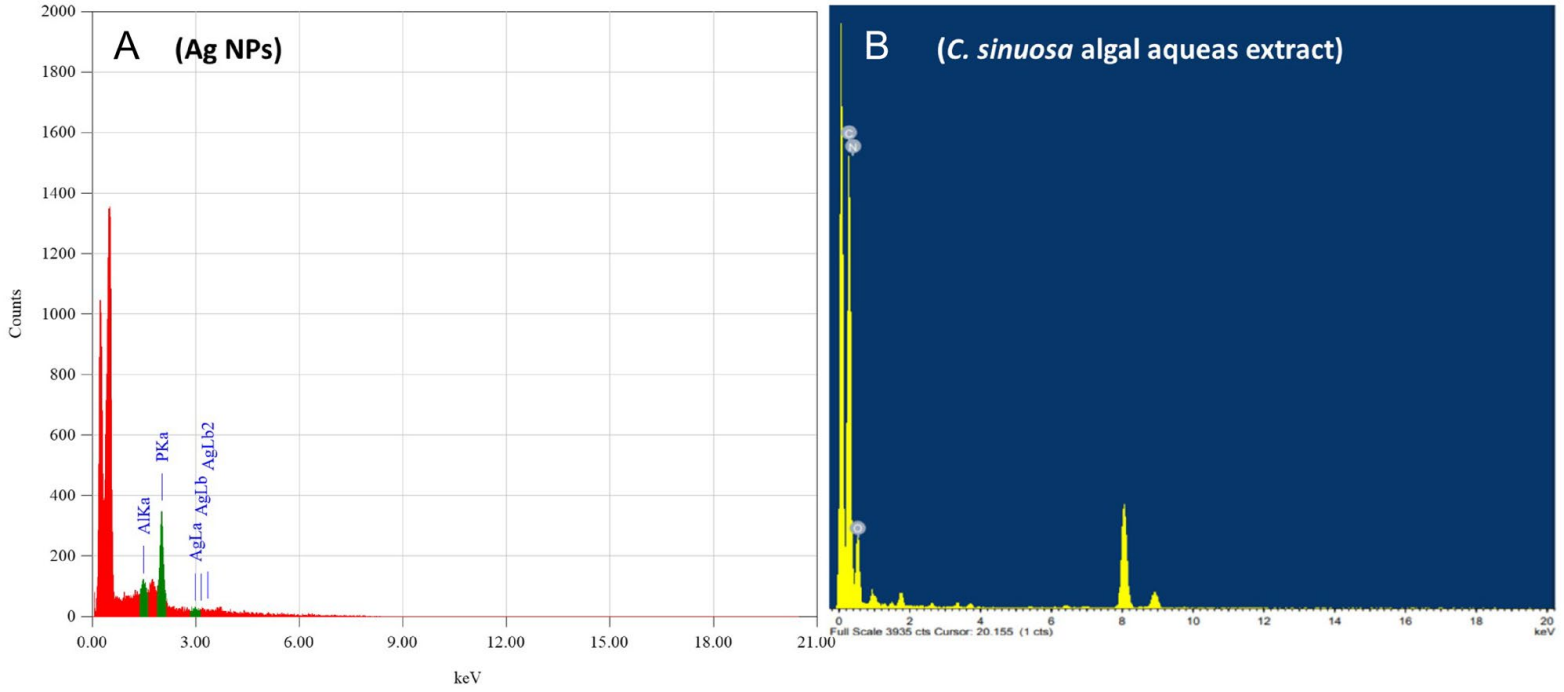
EDX analysis of (**a**) Ag NPs synthesized by using *C. sinuosa* extract and (**b**) for *C. sinuosa* algal aqueas extract.

**Figure 6:**
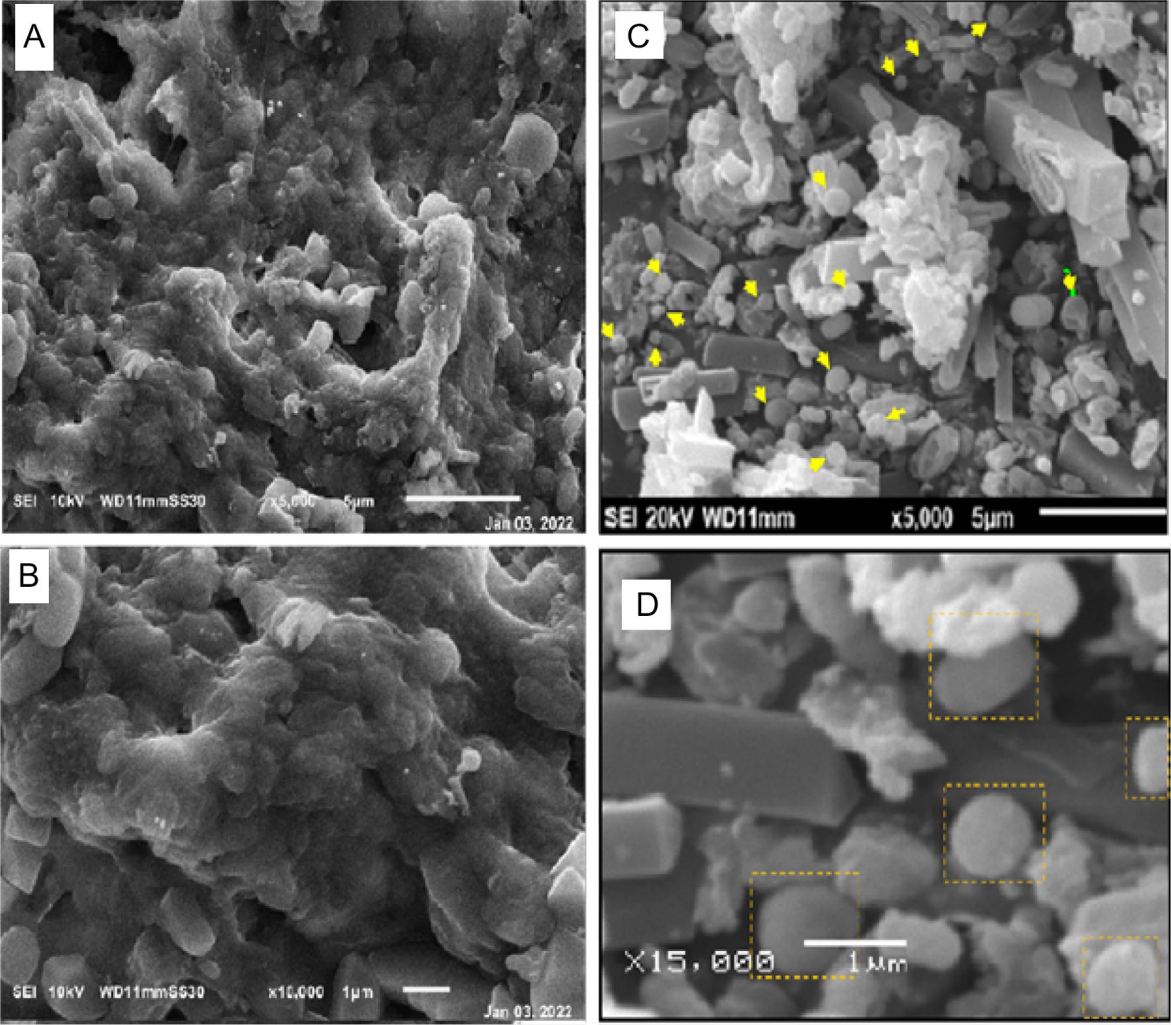
(**A**,**B**) Scanning Electron Microscope (SEM) micrograph of *C. sinuosa* algal aqueous extract and (**C**,**D**), Ag NPs synthesized by using *C. sinuosa* extract.

**Figure 7:**
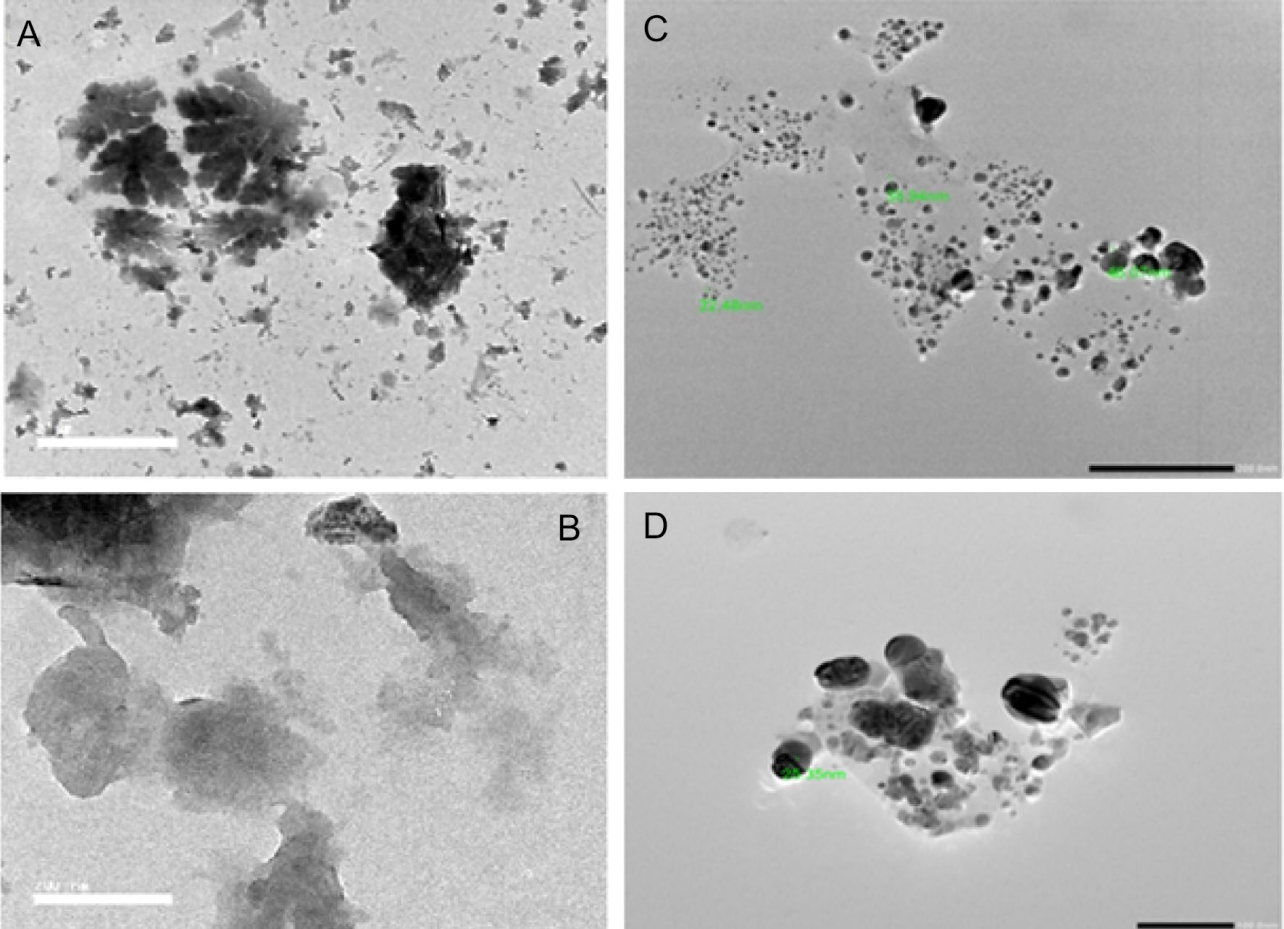
Transmission Electron Microscope (TEM) micrograph of (**A**,**B**) *C. sinuosa* algal aqueous extract and (**C**,**D**), synthesized silver nanoparticles from *C. sinuosa* extract.

The presence of the elemental silver signal of the Ag NPs and *C. sinuosa* algal extract in Fig. 5a,b was confirmed by energy dispersive spectroscopy (EDS or EDX). The presence of an optical absorption band at a 3 keV peak indicated the presence of pure metallic Ag NPs. SEM examination of colloidal spherical and hexagonal form Ag NPs revealed that they were in the 20–70 nm range in size, with some of them in the form of agglomerates, for evaluating the morphologies of biosynthesized nanoparticles. Biosynthesized nanoparticles have appeared as deposition on *C. sinuosa* extract (Fig. 6A,B). The Transmission Electron Microscope (TEM) was used to characterize and represent the distinguishing and size details of the established biosynthesized nanoparticles from *C. sinuosa* extract (Fig. 7A,B). The micrograph clearly shows that separate silver nanoparticles, as well as several aggregates, are present and they are spherical with the maximum diameter sizes of 22.8, 25.35, 33.94, and 46.07 nm for those prepared from the extracts of *C. sinuosa* extracts.

**Figure 8:**
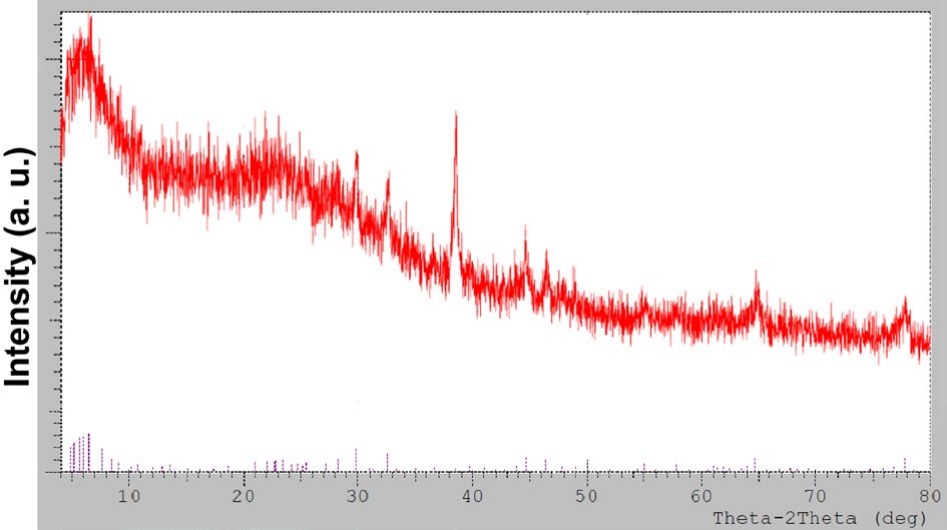
X-ray diffraction pattern of biosynthesized Ag-NPs by *C. sinuosa* extract.

The phase distribution, crystallinity nature, and purity of the biosynthesized nanoparticles from *C. sinuosa* extract are determined using an X-ray diffraction analysis of dry powders. Figure 8 depicts the XRD patterns of silver nanoparticles synthesized using *P. pavonica*, a marine brown alga. Several Bragg reflections are observed with 2θ values of 3.03° , 46.18° , 63.43° and 77.18° sets of lattice planes, which can be indexed to the 111, 200, 220 and 311 facets of silver respectively. The X–Ray diffraction pattern clearly shows that the silver nanoparticles formed in this synthesis are crystalline in nature, with a size of ~ 54 nm. Due to surface plasmon resonance, the metallic silver nanocrystals revealed an optical absorption peak of approximately 3 keV.

**Figure 9:**
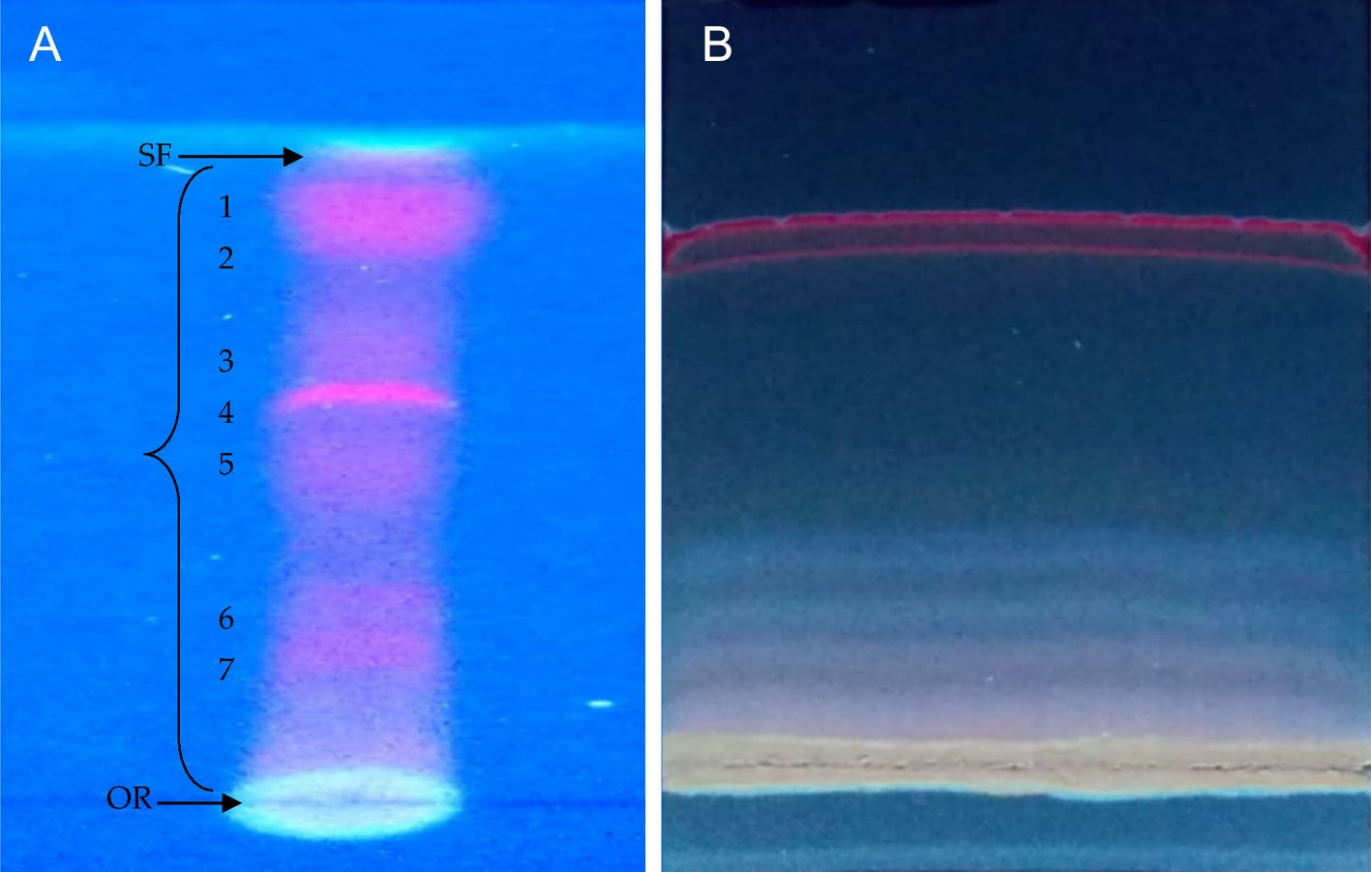
Thin layer chromatography of different *C. sinuosa* methylene chloride eluents (**A**) and preparative thin layer chromatography of the most effective eluent (**B**), the distance migrated by the solvent between the origin (OR) and solvent front (SF) is indicated near the brackets between those two boundaries.

### Evaluation of *C. sinuosa* and *C. mediterranea* extracts and their Ag NPs as nematicidal activity of *M. incognita *In vitro

In vitro study of the nematicidal activity of the C*. sinuosa and C. mediterranea* extracts' syntheses of nanoparticles that were tested against the J2S of the root-knot nematode *M. incognita* with concentrations (S = 100%, S/2 = 50%, S/3 = 75%, and S/4 = 25%) were compared with Nemacur (commercial nematicide) after 12, 24, and 72 h exposure time (Table 1).

**Table 1:** The effects of *Colpomenia sinuosa*, *Corallina mediterranea* macroalgal extracts and synthesized silver nanoparticles on J2s mortality % (M) of *Meloidogyne incognita* (MI) after 12, 24 and 72 h of exposure.

Treatment	Exposure time, number of alive J2S (L) and mortality % (M)						
	Con	12 h		24 h		72 h	
		L	M	L	M	L	M
(MI) (control)	–	12.2 a	-	17a	-	29a	-
MI + Nemacur	–	1.2 d	90.16	0.3f.	98.24	0.3 g	98.97
MI + *C. sinuosa* extract	S	8.7 b	28.69	4.2de	75.29	9.0c	68.97
MI + *C. sinuosa* SNPs	S	1.6 d	86.89	0.9f.	98.24	0.5 g	98.28
	S/2	2.4 d	80.33	1.5f.	91.18	1.06 g	96.34
	S/4	4.9 c	59.84	4.9dc	71.18	3.0f.	89.66
MI + *C. mediterranea* extract	S	11.0 a	9.84	6.3c	62.94	13.6b	53.10
MI + *C. mediterranea* SNPs	S	5.9 c	51.64	3.4e	80.00	3.7ef	87.24
	S/2	6.1 c	50.00	4.5de	76.47	5.0e	82.75
	S/4	9.0 b	26.23	8.5b	50.00	6.8d	76.55

* Data are means of 5 replicates. Means with the same letter(s), in each column, are not significantly different at P ≤ 0.05., L = Live. M = Mortality % = [(Total number of a live J2S in control—No. of alive J2S in treatment) / No. of Total alive J2S in control] × 100.

The data showed that MI + *C. sinuosa* treatments were more effective than *C. mediterranea* treatments, increasing *M. incognita* J2S mortality by 75.29 and 63% after 24 h, 68.97 and 53% after 72 h of exposure time, respectively. On the other hand, the treatment of MI + *C. sinuosa* NPs with concentration (S) was more effective than the other treatments at all concentrations with 87, 98.24 and 98.28% after 12, 24, and 72 h exposure time, respectively, which was comparable to the treatment of Nemacur as control and which was confirmed statistically.

### Evaluation of C. sinuosa and C. mediterranea extracts and Their Ag NPs as nematicidal activity of M. incognita In vivo (Pot experiment)

Table 2 shows the effects of *C. sinuosa* and *C. mediterranea* extracts and SNPs on the number of nematode galls (G), egg masses (EM), and eggs/egg-masses (E/EM) per tomato plant root infected with *M. incognita* (MI) after 60 days compared to Nemacur 400 EC. The results showed that the effect of *C. sinuosa* extract outperformed that of *C. mediterranea* in three parameters (G, EM, and E/EM), with reduction percentages of 53.24 and 8.65%, 73.24 and 52.11% and 80.40 and 49.44%, respectively.

**Table 2:** The effect of *Colpomenia sinuosa* (Cs), *Corallina mediterranea* (Cm) extracts, synthesized silver nanoparticles and Nemacur 400 EC on the numbers of nematode galls (G), egg masses (EM) and eggs/egg mass (Eggs) and Reduction % (R) in tomato crop infected with *M. incognita* (MI) after 60 days in a pot experiment Second item.

Treatment	Galls	*R%*	Egg Masses	*R%*	Eggs	*R%*
Control (MI)	92.5a ± 20.42	–	53.3 a ± 17.45	-	221.5 a ± 4.65	–
Plant + MI + Nemacur	3.4f. ± 4.18	96.32	4.75 bc ± 1.11	91.08	5.5 f. ± 52.93	97.52
Plant + MI + Cs	43.25c ± 24.30	53.24	14.3 bc ± 3.33	73.24	43.33 d ± 3.64	80.40
Plant + MI + Cs + SNPs	5.4 e ± 2.42	94.16	1.75 c ± 0.48	96.71	15.75 e ± 46.24	92.90
Plant + MI + Cm	84.5 b ± 26.92	8.65	25.5b ± 8.91	52.11	111.5 b ± 49.62	49.44
Plant + MI + Cm + SNPs	22.25 d ± 2.72	75.95	11.5 bc ± 6.51	78.40	100.57c ± 48.85	54.60

* Data are means of 10 replicates. Values followed by the same letter(s) are not significantly different at p ≤ 0.05.

On the other hand, the *C. sinuosa*-synthesized NPs and the Nemacur 400 EC treatments similarly reduced the number of *M. incognita* galls, egg-masses, and eggs/egg-masses (94%, 96%, and 96.71, and 91%, 92%, and 97%, respectively). For the three parameters, the efficacy of these two treatments surpassed that of the *C. mediterranea*-synthesized NPs and that of the normal extracts of both seaweeds, which showed statistically significant differences.

The effects of the algal extracts, biosynthesized AgNPs, and Nemacur 400 EC on the growth parameters of tomato plants infected with *M. incognita* after 60 days of nematode inoculation are shown in Table 3. The *C. sinuosa*-synthesized AgNPs had a positive effect on plant growth increasing shoot and root length by centimeters and the fresh weight of the shoot and root by grams. The root fresh weight increased by 94%, which was greater than the increase in all other treatments and the positive control. All treatments, on the other hand, had comparable effects on shoot fresh weight and were lower than the positive control.

**Table 3:** The effect of C*. sinuosa (Cs), C. mediterranea* (Cm) macroalgal extracts, synthesized silver nanoparticles and Nemacur 400 EC on some growth parameters of tomato plants infected with *M. incognita* (MI) after 60 days in a pot experiment and Increase % (I).

Treatment	Shoot system				Root system			
	Fresh weight (g)	*I*	Dry weight (g)	*I*	Fresh weight (g)	*I*	Dry weight (g)	*I*
Control (Healthy)	22.05 c ± 3.71	0	7.75 c ± 0.63	0	7.09d ± 0.82	0	2.52ab ± 0.32	0
Control (MI)	8.03 d ± 0.64	–	1.23 d ± 0.02	–	2.72e ± 0.34	–	0.99b ± 0.07	–
Plant + MI + Nemacur	27.39 a ± 2.56	24.22	9.05 c ± 0.58	16.8	9.00c ± 0.91	26.94	3.33a ± 0.25	32.14
Plant + MI + C.s	25.89 ab ± 1.43	17.41	11.80 b ± 0.35	52.3	9.75bc ± 0.5	37.52	3.24a ± 0.11	28.57
Plant + MI + C.s + SNPs	25.09 b ± 0.70	13.79	11.42 b ± 0.00	47.4	13.75a ± 2.3	93.94	2.87 a ± 0.05	13.89
Plant + MI + Cm	26.71 ab ± 1.12	21.13	13.95 a ± 0.03	80.0	13.22a ± 1.1	86.46	2.98 a ± 0.06	18.25
Plant + MI + Cm + SNPs	26.58 ab ± 1.25	20.54	14.45 a ± 0.77	86.5	11.23b ± 0.6	58.39	2.20ab ± 0.00	–

* Data are means of 10 replicates. Data expressed as mean ± SD. Values followed by the same letter(s) are not significantly different at p ≤ 0.05.

The *C. sinuosa* extract derived Ag NPs increased shoot dry weight by 47%, while the algal extract increased it by 52%; both were greater than the positive control (17%) but less than the *C. mediterranea* extract (80%) and Ag NPs (86%). Except for the *C. mediterranea*-derived AgNPs, all treatments had the same effect on root dry weight and were comparable to the positive control (Table 3).

The length of the plant shoots and roots did not change significantly after treatment or when compared to the negative control (Table 4). Conversely, tomato plants infected with *M. incognita* and treated with C.s. + biosynthesized AgNPs produced more fruits and flowers than that the other treatments (1.0 and 4.25, respectively).

**Table 4:** The effect of C*. sinuosa (Cs), C. mediterranea* (Cm) macroalgal extracts, synthesized silver nanoparticles and Nemacur 400 EC on some growth parameters of tomato plants infected with *M. incognita* (MI) after 60 days in a pot experiment.

Treatment	Length (cm)	Number of Fruit	Number of Flower	
	Shoot	Root		
Control (Healthy)	30.25a ± 4.59	13.25a ± 10.3	1a ± 0.408	2.57 ab ± 1.548
Control (MI)	23.72a ± 3.425	9.75 a ± 8.43	0b	0b
Plant + MI + Nemacur	28.75a ± 4.131	11.75a ± 10.08	0.5 ab ± 0.289	4 a ± 1.414
Plant + MI + Cs	30.5a ± 4.592	12.5 a ± 10.24	0.25 b ± 0.25	2.5 ab ± 1.041
Plant + MI + Cs + SNPs	31a ± 5.452	15.75a ± 11.2	1.0a	4.25 a ± 1.548
Plant + MI + Cm	23.25a ± 3.082	9 ± 9.05a	0.5 ab ± 0.289	1.75 ab ± 0.629
Plant + MI + Cm + SNPs	25.25a ± 4.708	14 ± 8.4a	0b	0b

Data are means of 10 replicates. Data expressed as mean ± SD. Values followed by the same letter(s) are not significantly different at p ≤ 0.05.

Thin-layer chromatography revealed that hexane: methylene chloride: ethyl acetate (1: 0.5: 0.5 v/v) was the most effective *C. sinuosa* methylene chloride eluent (Fig. 7A). This eluent was fractionated into four fractions (Fig. 7B), each of which was tested separately for its effect on *M. incognita* (Table 5). The third fraction was the most effective, with 88% mortality after 12 h and absolute mortality (100%) after 24 h and 72 h of exposure, which was comparable to the positive control for all three time-periods.

**Table 5:** The effects of *C. sinuosa* methylene chloride eluent fractions on J2S mortality % (M) of *Meloidogyne incognita* (MI) after 12, 24 and 72 h of exposure.

Treatment	(J2S mortality %)					
	12 h		24 h		72 h	
	L	M (%)	L	M (%)	L	M (%)
(Neg. control) (MI)	3.2 a	–	4.2 a	–	5.2 a	–
(Pos. control) MI + Nemacur	0.2 c	93.75	0.2 bc	95.24	0.00 b	100
MI + The first fraction	0.6 b	81.25	0.6 b	85.71	0.4 b	92.31
MI + The second fraction	0.6 b	81.25	0.2 bc	95.24	0.0 b	100
MI + The third fraction	0.4 bc	87.50	0.0 c	100	0.0 b	100
MI + The fourth fraction	0.6 b	81.25	0.1 c	97.62	0.0 b	100

Data are means of 5 replicates. Values followed by the same letter(s) are not significantly different at p ≤ 0.05.

**Figure 10:**
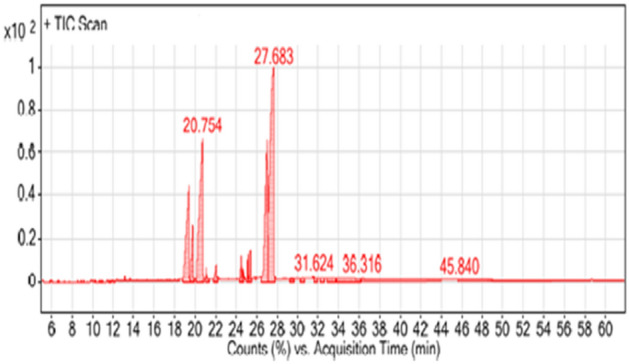
GC–MS chromatograph of the most effective fraction (the third fraction) of *C. sinuosa* methylene chloride eluent.

The GC–MS results of the most effective fraction (the third fraction) of *C. sinuosa* methylene chloride eluent (Hexane: methylene chloride: ethyl acetate; 1.5: 0.5: 0.5 v/v) revealed the presence of seven bioactive constituents, with five major compounds (Table 6 and Fig. 10). They are primarily dibutyl phthalate and its two isomers (11.68, 4.18 and 22.42%); methy methyltetradecanoate (0.76%); palmitic acid (1.34%); 1-propene-1, 2, 3-tricarboxylic acid, tributyl ester and its two isomers (1.16, 1.04 and 1.25%); and tributyl acetyl citrate, and one isomer (15.57 and 40.60%).

**Table 6:** Major phytocompounds detected in the most effective fraction of methylene chloride eluent by GC–MS analysis.

Peak	Retention time (min)	Area %	Detected compound	Formula	MolecularWeight	Probability (%)	Structure
1	13.172	-	Isophoronediisocyanate	C12H18N2O2	222	71.6	
2	13.695	-	2-Methylenecholestan-3-ol	C28H48O	400	45.1	
3	19.395	11.68	Dibutyl phthalate	C16H22O4	278	19.2	
4	19.782	4.18	Dibutyl phthalate	C16H22O4	278	19.2	
5	20.754	22.42	Dibutyl phthalate	C16H22O4	278	12.2	
6	21.098	0.76	methyl 12-methyltetradecanoate	C16H32O2	256	14.4	
7	22.082	1.34	Palmitic acid (Syn. Hexadecanoic acid)	C16H32O2	256	30.8	
8	24.541	1.16	1-Propene-1,2,3-tricarboxylic acid, tributyl ester	C18H30O6	342	93.2	
9	25.174	1.04	1-Propene-1,2,3-tricarboxylic acid, tributyl ester	C18H30O6	342	92.0	
10	25.414	1.25	1-Propene-1,2,3-tricarboxylic acid, tributyl ester	C18H30O6	342	92.4	
11	27.056	15.57	Tributylacetylcitrate	C20H34O8	402	93.5	
12	27.683	40.60	Tributylacetylcitrate	C20H34O8	402	88.2

## Discussion

The purpose of this research is to evaluate green AgNPs as a potential replacement for hazardous and environmentally damaging chemical nematicides^2,5,21,58^. The obtained silver nanoparticles were aggregated into anisotropic Ag particles, as evidenced by the SEM micrograph (magnified at 5000$\times$). Pal et al.^59^ also reported that the Ag particles were aggregated into nanorods with an average edge length of more than 100 nm. The TEM images, on the other hand, revealed monodispersed AgNPs with spherical shapes of less than 40 nm in diameter. The particle size was increased by up to 4000 nm. These findings were strikingly similar to those obtained by SEM and FT-IR. The crystalline nature of the nanoparticles is demonstrated by selected area electron diffraction patterns with circular or rod spots, where the average particle size in the current study was found to be 22.48, 33.94 and 46.07 nm, as shown in the size distribution graph. Similarly, Devi and Bhimba^60^ reported silver nanoparticles with sizes ranging from 20 to 56 nm prepared with *Ulva lactuca*, whereas Abdellatif et al.^61^ reported Ag NPs with smaller sizes ranging from 8 to 19 nm prepared with *Turbinaria turbinata*. The FT-IR analysis spectrum for the synthesized nanoparticles revealed sharp absorbance between 440 and 4000 cm^−1^, with distinct peaks 3915- 3900–3751, 3421, 2928, 1637–1533, 1386–1327-1228, 1072, and 532–474. Thus, FT-IR analysis showed the multifunctionality of nanoparticles synthesized from *C. sinuosa* extract, with proteins, phenols, and other groups present in the aqueous extract of the *C. sinuosa* responsible for the reduction of Ag + to AgO and the stabilization of the synthesized AgNPs.

The bioassay results showed that the treatment with *C. sinuosa* synthesized NPs was the most effective and comparable to the full concentration commercial pesticide Nemacur 400 EC in eliminating juvenile *M. incognita* after 72 h of exposure, though the effectiveness decreased with lower NP concentrations. These findings extended to the reduction of *M. incognita* three parameters (the number of galls, EM and egg/egg mass). Conversely, the algal extract alone was not as effective as the synthesized AgNPs, and its nematicidal activities were lower than that of Nemacur 400 EC. Chemical nematicides are typically more effective than other strategies, but they have caused significant environmental problems due to their toxic residues and their use is frequently severely restricted^62^. Algae were considered to be a good nematode control alternative^63^ while NPs, such as nanosilver, have been recently adopted for controlling plant pathogens, including nematodes^36,64^. Laboratory experiments have shown that 2 to 4 days of exposure time is required to reduce J2S counts in root-knot nematodes when comparing the effect of chemically synthesized AgNPs on J2s^22^. Our results are consistent with those of Abdellatif et al.^61^, who used AgNPs incorporated into algal extracts from *Ulva lactuca* and *Turbinaria turbinata* to test the nematicidal effect on infected eggplants (*Solanum melongena* cv. Login) in greenhouses. They found that AgNPs at 12.75 mg/100 ml concentrations from both algal species were as effective as chemical pesticides at controlling root-knot nematodes in eggplants, while causing no phytotoxicity in the eggplants. The beneficial effect of NP was attributed by Abdellatif et al.^61^ to their association with compounds from algal extracts, which contain many major and minor nutrients required by plants, including many organic compounds such as auxins, gibbrellins, and ethylene and betaine precursors^65^. PNs' mode of action, according to Abdellatif et al.^61^, is non-specific and associated with the disruption of multiple cellular mechanisms, including membrane permeability, ATP synthesis, and oxidative stress response^66^. Abdellatif et al.^61^ went on to say that combining NPs with algae that supplement the NPs nematicidal effect increases the NPs effectiveness. It is worth noting that the United States Environmental Protection Agency recommends a maximum allowable level of elemental silver in drinking water for short-term (1–9 days) individual consumption of 2.5 g/ml^67^. Consequently, the concentration of Ag in AgNO3 NPs applied in our study was lower than this dose (1.08–1.35 µg/ml). However, lipid-soluble extracts of marine macroalgae extensively researched as a potential source of novel pharmacological compounds. Several organic solvents were used to screen algal compounds for antibacterial and nematicidal activity^35,37,61,68^. Methylene chloride was used in this study to extract bioactive compounds from two tested macroalgal species, with *C. sinuosa* algal extract having higher anti-nematode activity against *M. incognita* than *C. mediterranea*. Five major compounds were identified as potent organic compounds in the current study: dibutyl phthalate and its two isomers, methyl 12-methyltetradecanoate, palmitic acid, 1-propene-1, 2, 3-tricarboxylic acid, tributyl ester and its two isomers and tributyl acetylcitrate and its one isomer. One of these natural bioactive compounds was a fatty acid and others were esters, tetracarboxylic acids and phthalate derivatives, in addition to isophorone diisocyanate and 2-Methylenecholestan-3-ol, which were detected by the GC–MS of the most effective fraction of methylene chloride. Rizvi et al.^69^ reported that iso-phorone diisocyanate has antibacterial properties, while Shareef et al.^70^ noticed that 2-methylene choles-tan-3-ol has cytotoxic properties. Palmitic acid was found to have antioxidant, nematicide, pesticide, antifouling, antibacterial, anti-inflammatory, and antifungal activity^71^. Nematicide activity has been reported for Methyl 12-methyltetradecanoate^71^. The bioactive compound 1-propene-1, 2, 3-tricarboxylic acid, tributyl ester is known as aconitic acid. Trans-aconitic acid (TAA) is an isomer of Cis-aconitic acid (CAA). Cuiying et al.^72^ discovered that (TAA) showed activity against the plant-parasitic nematode *M. incognita*, whereas CAA had a much weaker nematicidal effect. According to the findings of this study, *C. sinuosa* is a producer of aconitic acid, which has nematicidal activity. On the other hand, dibutyl phthalate is used as an ectoparasiticide^34^. Many studies, however, have reported phthalate derivatives' nematicidal activities. El-Deen et al.^35^ used GC–MS to analyze the algal ethanolic extract of *Ulva fasciata* as a promising nematicide, which revealed the presence of organic component such as bis (2-ethylhexyl) phthalate at 63.75% and diethyl phthalate at 18.46%. Khan et al.^37^ investigated the biochemical potential of seaweed in two different solvents viz., water and methanol at ratios of 2.5, 5 and 10%. After 72 h, methanol extract (10%) of *Colpomenia sinuosa* recorded 82 ± 2.84% egg hatching and 91 ± 1.76% larval mortality.

## Conclusions

The nematicide activity of silver nanoparticles synthesized from the brown alga *Colpomenia sinuosa* outperformed that of the commercial Nemacur 400 EC and the algal extract of the same species. As a result, it can be used to control of *Meloidogyne incognita* as an alternative to chemical nematicides. However, additional research on purification and isolation of potent bioactive compounds is required to determine which one is the most effective. Using such a technique in root-knot nematode management could significantly improve new trends that are safe, eco-friendly, and effective against the root-knot nematodes control program. So, more research is needed to develop bio fabricated green nanoparticles that are toxic and kill nematodes while also having biodegradation modes of action before they can be recommended for field application and IPM programs against plant-parasitic nematodes on various crops.

## Data Availability

The data utilized to support the findings of this research are included within the article.
